# Specific Mass Activity and Surface Activity of Platinum Electrically Connected with CNTs in the Oxygen Reduction Reaction

**DOI:** 10.3390/membranes13100832

**Published:** 2023-10-15

**Authors:** Andrey Nechitailov, Anna Krasnova, Nadezhda Glebova

**Affiliations:** Ioffe Institute, St. Petersburg 194021, Russia

**Keywords:** ORR, specific mass activity, platinum surface activity, carbon nanotubes, fuel cell

## Abstract

This paper presents a study of the platinum activity in the ORR in a hydrogen polymer electrolyte membrane fuel cell with electrodes containing multi-walled CNTs in a wide range of compositions and conditions. The data of the comparative analysis of the platinum activity on a fraction of Nafion in the electrode, the composition of the oxidizing agent (oxygen, air), pressure, and temperature are provided. The reasons for the dependence of the platinum surface activity on the component composition of the electrode are considered. Specific mass activity and surface activity of platinum in the ORR in MEA with the electrodes with CNTs depend on the ionomer/platinum ratio. Both dependences have a maximum at the level of the 25% Nafion fraction. The maximum appears as a result of an optimal structure formation, which ensures the fullest use of the platinum surface and minimal concentration overvoltages. Specific mass activity and surface activity of platinum for the sample with 34% CNTs at *T* = 60 °C and excessive pressure of *p* = 2 atm amount to 0.46 A/mg and 0.72 mA/cm^2^, respectively.

## 1. Introduction

Researchers and manufacturers of fuel cells with proton exchange membranes (PEMFC) are especially interested in issues related to electrocatalytic activity [1,2,3,4,5,6].

The use of various carbon-based materials and certain compounds as metal catalyst supports was analyzed in the study presented by Wang et al. [1].

It was noted that the commercialization of PEMFC technology is strongly hindered by challenges associated with the kinetics of the oxygen reduction reaction (ORR) at the cathode and a high cost of Pt-based cathodic catalysts (with the latter currently accounting for more than 55% of the total PEMFC amount). The issues of overcoming the limited stability of the modern Pt/C, Pt, and Pt alloy catalysts supported on modified carbon materials have attracted considerable interest in recent years. The study represents a systematic and comprehensive analysis of the modern cathodic PEMFC catalysts from Pt and Pt alloys in terms of selection and design of materials, synthesis methods, and structural features. The requirements for the catalytic system were formulated, among which high electrode activity and durability are the main ones. The trend of recent years is a wide use of modified carbon materials to increase the electrocatalytic activity of the metal catalyst in the ORR. In this case, synergism in the catalysis involving functional groups of carbon and metal catalysts (Pt, Pd, and various alloys with other metals) is used [2,3,4,5,6]. Vinayan et al. [2] studied the role of functionalized multi-walled carbon nanotubes (MWCNTs) decorated with platinum nanoparticles (Pt/*f*-MWCNT) and platinum–cobalt alloy nanoparticles (Pt_3_Co/*f*-MWCNT) in ORR PEMFC. The electrocatalysts were synthesized using two methods: the traditional sodium borohydride reduction method and the modified polyols reduction method. The modified polyols reduction method provides better homogeneity of variances, higher loading, and optimal particle size of Pt and Pt_3_Co alloy nanoparticles compared to the usual sodium borohydride reduction method.

Pt_3_Co/*f*-MWCNT synthesized by the modified polyols reduction method provides high performance with the highest power density of 798 mW/cm^2^ at *T* = 60 °C. The increased catalytic activity of Pt_3_Co/f-MWCNT towards the ORR is explained by the uniform distribution and optimal particle size of Pt_3_Co alloy nanoparticles on the *f*-MWCNT surface. Chandran et al. [3] used a Pd_3_Co/NG (N-doped graphene) catalyst to replace platinum. Heydari et al. [4] applied an efficient approach to prepare nitrogen-doped Pt (Pt/N-rGO) graphene nanoparticles. Graphene nanocomposites doped with nitrogen (N-rGO) were obtained by pyrolysis of graphene oxide/polyaniline composites in a nitrogen atmosphere. To characterize the morphology and microstructure of the prepared catalysts, powder X-ray diffraction, FTIR spectroscopy, Raman spectroscopy, X-ray photoelectron spectroscopy, and transmission (TEM) and scanning electron microscopy (SEM) were used. Images obtained by TEM and element mapping show that metal nanoparticles are more evenly dispersed on the surface of nitrogen-doped graphene than on other carriers, and Pt nanoparticles are dispersed without any aggregation. The catalytic activity and durability of the catalysts were evaluated by various electrochemical methods. Increased electrocatalytic activity was obtained in the case of Pt/N-rGO with optimized composition and nanostructure in comparison with the undoped Pt/rGO and commercial Pt/C catalysts. The maximum specific power of the membrane electrode assembly (MEA) for Pt/N-rGO was 1.4 times that of MEA made from commercial Pt/C (20% Pt). Tellez-Cruz et al. [5] described the use of CNT, nitrogen-doped graphene, and various forms of sulfur-doped carbon to increase the activity and durability of the catalyst.

The performance evaluation (material selection, chemical reaction modeling, and polarization curves), durability prediction (state of health, fault diagnostics, and remaining useful life), and application monitoring of fuel cells were the focus of a systematic review conducted by Ming et al. [6]. Traditional machine-learning (ML) and deep-learning method comparisons are reviewed, and a comparison between ML and integrated physics simulations is also drawn. Eventually, the extent of machine-learning techniques used in fuel cells is discussed, and prospects for further studies on ML applications in fuel cells are noted.

The reported data on platinum’s catalytic activity in the ORR vary greatly, sometimes by up to three orders of magnitude. The data on the catalytic activity of platinum in the ORR for various catalysts at 0.9 V are provided by Neyerlin et al. [7]. Data are given for MA within the range of 0.01 to 0.25 A/mg (Pt) and SA within the range of 0.32 *×* 10^−4^ to 3.9 *×* 10^−4^ A/cm^2^ (Pt). Kriston et al. modeled mass activity of platinum [8]; it was noted that the porosity, agglomerate size, and Nafion layer thickness influence the mass and specific activities by shifting the double Tafel slope asymptotic solutions to more positive potentials, and, consequently, the mass and specific activities slightly decrease even at 0.9 V (*iR*-free). However, the loss of the validity of the Tafel approximation is clearly indicated by the increased Tafel slope from the theoretical value (0.07 V/dec). Banham et al. [9] studied the activity at ultra-small loads of Pt, and the high value of carbon support was noted.

Xiao et al. [10] reported that a promising strategy is alloying Pt with transition metals where synergistic effects and strain inducted on Pt ad-atoms can lead to unprecedented activity. Herein, the authors report a facile and simple one-pot synthesis method, to synthesize carbon-supported PtCuFe nanoparticles surpassing the Department of Energy (DOE) USA technical requirement of an ORR catalyst for PEMFC application. The catalyst shows an ORR mass activity of 0.99 A/mg (Pt) at 0.90 V vs. RHE and records 2.9 times enhancement over current commercial TTK catalysts. At the same time, the use of such rather active metals as iron and copper is connected with the hazard of “poisoning” with Nafion as a result of replacement of H^+^ ions by metal cations. Jongmanwattana et al. [11] prepared the sample of Pt/graphene electrocatalyst using an aging time of 2.0 h, which yielded the highest SA, at around 0.764 mA/cm^2^.

As we can see, there is a considerable scatter in the platinum activity values found in the scientific literature. This is due to the fact that the platinum activity is strongly influenced by many factors, such as the structure, component composition of the electrode, platinum loading, and the interphase boundary, etc.

One of the current (by 2025) DOE targets for mass activity of platinum in the ORR is 0.44 A/mg. It should be noted that this activity is not a separately declared indicator, but depends on a set of other MEA characteristics, such as durability and cost price. We believe that this is an important point when comparing the data of different authors, and it also explains such a large difference in the published data.

It should be mentioned that most of the works describe the use of various carbon forms as metal catalyst supports. The use of CNT as an independent component of a composite electrode is attractive because it provides greater flexibility in controlling the composition and structure of the electrode. Furthermore, the absence of direct contact of CNTs with platinum reduces their electrochemical corrosion. For example, Kanninen et al. [12] observed significant efficiency losses for Pt/CNT-based electrodes, which, as the authors note, are probably associated with the degradation of the catalytic layer during corrosion. SEM images of the electrodes before and after corrosion showed that the Pt/CNT-based catalyst forms very thick and porous catalyst layers, which after corrosion, only half of their original thickness remains, while the Vulcan-based catalyst forms a thin layer that was far less corroded.

In this work, a structurally new material has been investigated. The material consists of a traditional catalyst: Pt nanoparticles on carbon black (Pt/C), connected by numerous electrical contacts with MWCNTs. Such a catalyst is of interest because of the combination of a highly porous structure and high Pt activity due to the influence of CNTs along with relative stability. However, there are practically no publications on the study of such structures. Thus, there is a gap in the study of catalysts containing individual CNTs connected to Pt only by electrical contacts. The kinetic parameters of the ORR via rotating disk electrode were studied in our previous work [13]. The effect of CNTs on Pt sites in the ORR was explained by the effect of oxygen-modified CNTs on the content of surface oxide (PtO); in the presence of CNTs, the PtO content is lower. The aim of this work was to study the effect of CNTs, used as an independent component, on the activity of platinum in the ORR as part of MEA, i.e., in real devices.

## 2. Materials and Methods

### 2.1. To Produce MEA, the Following Components Were Used

E-TEK platinized carbon black (40% Pt) [14] was used as an active catalyst component. The material consists of Pt nanoparticles with a size of approximately 3 (2.8) nm deposited on a carbon support: carbon black of the Vulcan-XC-72 type. Platinum is in the elemental (metallic) state and has an oxidation state of 0. Since there are mechanical contacts between platinized soot and carbon nanotubes, the metallic platinum nanoparticles are in electrical contact with the carbon nanotubes.

Multi-walled CNTs of the Taunit MD brand (NanoTechCenter LLC, Tambov, Russia) with a high length/diameter ratio reaching the value of >1000 and high porosity > 80% were used. The outer diameter was 8–30 nm, the inner diameter 5–15 nm, length ≥ 20 μm, bulk density ≥ 0.025–0.060 g/cm^3^, and specific surface ≥ 270 m^2^/g. Nafion solution DE2020 (DuPont™, Wilmington, DE, USA) was used. Isopropanol (99.80%, ECOS-1 JSC) was used. Deionized water with resistivity at room temperature ρ ≥ 18 MOhm×cm was used. MEAs were fabricated using an analog of the Nafion 212-type membrane called the MF4-SK-type (OJSC “Plastpolymer”, St. Petersburg, Russia), which is 50 μm thick. Before use, CNTs were subject to additional treatment in an ultra-high pure nitric acid solution diluted with deionized water at a volume ratio of 1:1 for deep purification from metal impurities. The treatment was carried out at a temperature of *T* = 100 °C for 10–15 min with stirring. After that, the suspension was filtered and washed with deionized water five times until the wash water had a neutral reaction.

### 2.2. EDX

Energy-dispersive X-ray elemental microanalysis was performed on a Quanta 200 scanning electron microscope (FEI Company, Dawson, NE, USA) equipped with an EDAX microprobe attachment.

### 2.3. Microscopic Studies

The target-oriented approach was utilized for the optimization of the analytic measurements [15]. The samples were secured on a 3 mm copper grid and fixed in a grid holder before measurements were taken. The samples’ morphology was studied using a Hitachi SU8000 field-emission scanning electron microscope (FE-SEM) (Hitachi High-Technologies Corporation, Tokyo, Japan). At 30 kV accelerating voltage, images were acquired in bright-field STEM mode.

### 2.4. Preparation of Catalyst Inks

The technological procedures for preparing the electrode material’s dispersion included two stages: mechanical and ultrasonic dispersion of the mixture of precise samples of components in the i-propanol–water mixture. The volume ratio of the i-propanol–water liquid components was in the range of 1:4–1:20. The ratio of the solid to liquid phases in the final dispersion in this case was in the range of 1:60–1:120. Samples with CNTs were prepared using a method assuming preliminary coagulation of Nafion from its solution in the liquid phase, followed by its introduction into the electrode structure [16,17]. For coagulation, a commercial Nafion dispersion with the required concentration was diluted with water in a volume ratio of 1:1 prior to its addition to the dispersion.

Mechanical dispersion was performed in a Milaform MM-5M magnetic stirrer (Milaform-service, Neftekamsk, Russia) with a velocity of core rotation of approximately 400 rpm (the core was enclosed into a plastic casing) to obtain a visually homogeneous mixture (without visible blobs) (~0.5 h). A Branson 3510 ultrasonic bath was then used for a subsequent ultrasonic dispersion process that lasted 40–100 h in order to obtain a homogeneous dispersion that did not separate after one minute.

### 2.5. MEA Preparation

MEA was prepared by applying thin dispersions of the components in the mixture of i-propanol–water (catalyst inks) on the surface of the proton-conducting membrane consequently from both sides. To do this, the membrane was thermostated at *T* = 85 ± 5 °C, and the area of electrode material applying 1 × 1 cm^2^ in size was limited by the stainless-steel mask.

### 2.6. XRD Studies

An X-ray diffractometer X’Pert (Malvern Panalytical Ltd., Malvern, UK) with Cu K radiation (λ = 1.54060 nm) was used to collect the X-ray diffraction (XRD) pattern. The software HighScore Plus 3.0.5 was used to process the diffraction pattern. The size and microstrains of the crystallites were refined and determined using the Rietveld method.

### 2.7. Testing of Samples

Testing of electrocatalysts in the MEA was carried out according to DOE protocols [18] as follows.

The MEA was placed in a standard electrochemical cell (FC-05-02, ElectroChem Inc., Wo-burn, MA, USA) with graphite current collectors with the following characteristics: temperature maintenance that ranged from room temperature to *T* = 180 °C, gas overpressure *p* = 0–2 atm, and electronic resistance less than 10 mΩ [19]. Toray 060 standard carbon paper was used as the gas diffusion layer. Before starting the main measurements, the MEA was activated as described in our previous work [20].

A humid O_2_/H_2_ or air/H_2_ system was used to prevent the membrane from drying out. Gases from the generators were bubbled through deionized water and supplied to the electrodes.

The potential of the electrode under study was refined by the formula:

*E = E_set_ + It × R*, (1)

where *E_set_*—the set value of the potential; *It × R*—the ohmic potential drop equal to the product of the current strength by the resistance (*R*) of the MEA.

The electrochemically active surface of platinum (ESA) was measured by hydrogen desorption according to a well-known method [21,22] based on the measurement of the charge passed for hydrogen desorption from the platinum surface in hydrogen area in *i–V* curves in the N_2_/H_2_ system.

The Nyquist plot was registered in the N_2_/H_2_ system with the Z500X+AX500PL device in the frequency range of 500 kHz−0.1 Hz at a voltage close to the open circuit voltage (polarization 0−100 mV) at an AC voltage amplitude of 8 mV and a charge-transfer resistance of *R* > 80 Ω.

The principle of formal kinetic analysis based on the Arrhenius equation was used to evaluate the kinetics of oxygen reduction [23].

The analysis of Arrhenius dependencies allows us to not only calculate the kinetic parameters of the reaction (*Ea* and *A*), but also to evaluate the change in the reaction mechanism. In the current case, when the potential changes, the apparent activation energy changes (the slope changes). This indicates the appearance of so-called mixed kinetics, when the rates of other reactions begin to contribute to the resulting reaction rate. With a small overvoltage (high potential), as is known, the reaction rate is determined by the rate of actual charge transfer (kinetic current). When the overvoltage increases (potential decreases), diffusion restrictions (concentration overvoltage) are added [24].

### 2.8. Calculations

The ESA was calculated from the ratio:

*S_Pt_ = Q_des_/*210, (2)

where *S_Pt_*—Pt ESA, cm^2^; *Q_des_*—charge spent on hydrogen desorption, µC; 210—coefficient relating the charge to the surface, µC/cm^2^.

The Pt loading was gravimetrically calculated by the ratio:

*G_Pt_* = *M_CL_* × *N* × 0.4, (3)

where *G_Pt_*—Pt loading in the electrode, mg/cm^2^; *M_CL_*—catalytic layer weight, mg; *N*—proportion of Pt/C in the catalytic layer; 0.4—proportion of Pt in E-TEK.

The specific mass activity (MA) of Pt in the ORR was calculated from the known relation:

MA *= J@E/G_Pt_*, (4)

where MA—Pt MA at a potential of *E* = 0.9 V vs. NHE, A/mg (Pt); *J@E*—current density of the ORR at a potential of *E* = 0.9 V vs. NHE, A/cm^2^; *G_Pt_*—Pt loading in the electrode, mg/cm^2^.

The Pt surface activity (SA) in the ORR was calculated by the ratio:

SA = (*J@E × S_CL_*)/*S_Pt_*, (5)

where SA—Pt SA at a potential of *E* = 0.9 V vs. NHE, mA/cm^2^; *J@E*—current density of the ORR at a potential of *E* = 0.9 V vs. NHE, mA/cm^2^; *S_CL_*—visible electrode surface area, 1 cm^2^; *S_Pt_*—Pt ESA, cm^2^.

The principle of formal kinetic analysis based on the Arrhenius equation was used to calculate the kinetics of oxygen reduction:

*k* = *Ae*^−*Ea/RT*^, (6)

where *k*—reaction rate constant; *A*—pre-exponential factor; *Ea*—activation energy; *R*—gas constant; *T*—absolute temperature.

The following relation is obtained by taking the logarithm of relation (6):

*ln(k) = ln(A) − Ea/RT*, (7)

or

*ln(k) = ln(A) − (Ea/R) × (*1*/T)*, (8)

When the reaction rate constant is expressed in terms of the current density *J*, we obtain:

*ln(J) = ln(A) − (Ea/R) × (*1*/T)*, (9)

When plotting the dependencies of *ln(J)* on the inverse temperature (1/*T*), kinetic (Arrhenius) dependences of the logarithm of the reaction constant (rate) on temperature are obtained. In the case of a first-order reaction, these are straight lines. As can be seen from the relation (9), the slope tangent of the straight lines is equal to the ratio of the activation energy to the universal gas constant: *tg*α = −*Ea/R*, i.e., the greater the slope, the greater the activation energy. The intersection of the straight line with the ordinate axis corresponds to a pre-exponential multiplier.

## 3. Results and Discussion

EDX analysis showed that CNTs contain impurities, which were removed by treatment in nitric acid. Table 1 shows that after acid treatment, oxygen content increased, and the total content of metal impurities decreased significantly.

Seven types of MEA samples with electrodes of various compositions were fabricated. The amount of CNTs varied from 10 to 45%. Table 2 represents the features of studied MEA samples.

**Figure 1:**
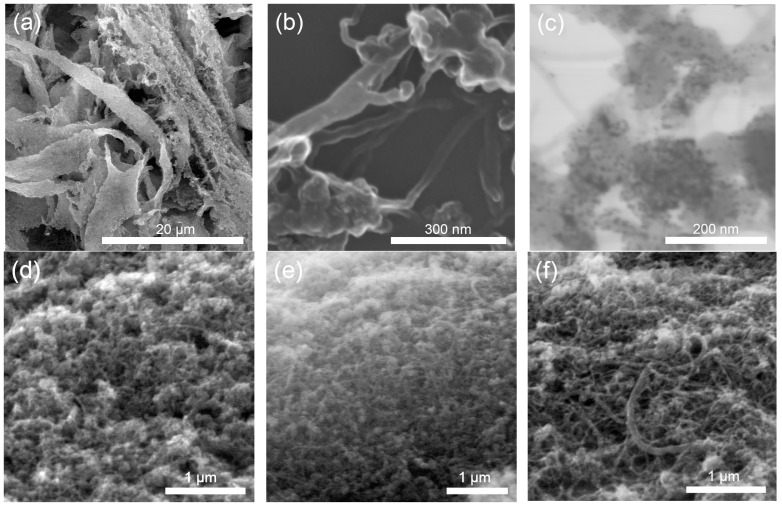
SEM (**a**,**b**) and TEM (**c**) micrographs of the electrode with 30% CNTs at various magnifications; SEM micrographs of the electrode with various CNTs content: 10% (**d**), 20% (**e**), and 48% (**f**).

Micrographs of electrodes at various magnification are shown in Figure 1a–c. Micrographs of the electrodes with various CNTs content are presented in Figure 1d–f.

The figure shows that the electrode has large pores of micron size (Figure 1a) and fine structure (Figure 1b). Pt particles are presented in Figure 1c. Such morphology ensures intensive mass transfer (due to high porosity and presence of transport pores) in combination with efficient use of the platinum surface. Micrographs of electrodes with different CNT content (Figure 1d–f) illustrate the effect of CNTs on the electrode structure. The electrode with 48% CNTs has more macropores (Figure 1f).

**Figure 2:**
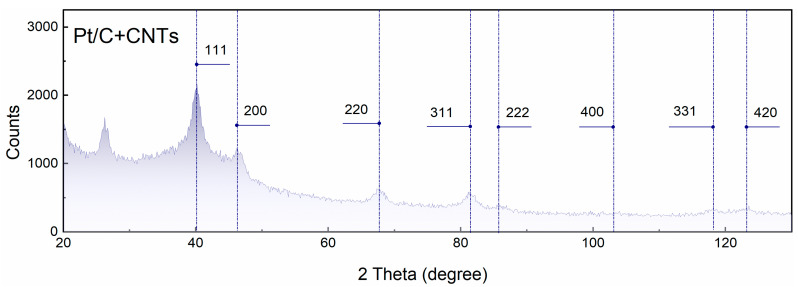
X-ray diffraction pattern of Pt/C+CNTs electrode. Blue lines correspond to the diffraction peaks at about 40°, 46°, 68°, 81°, 86°, 104°, 118°, and 123° due to Pt (111), (200), (220), (311), (222), (400), (331), and (420) reflections.

Figure 2 depicts the results of the XRD analysis of the MEA_CNT-30 crystal structure. The face-centered cubic structure of Pt (ICDD 03-065-2868) was confirmed by the diffraction peaks at approximately 40°, 46°, 68°, 81°, 86°, 104°, 118°, and 123°, which are attributed to Pt (111), (200), (220), (311), (222), (400), (331), and (420) reflections, respectively. The intensive peak at 2θ = 26.8° conforms to graphite, plane (002) [25].

**Figure 3:**
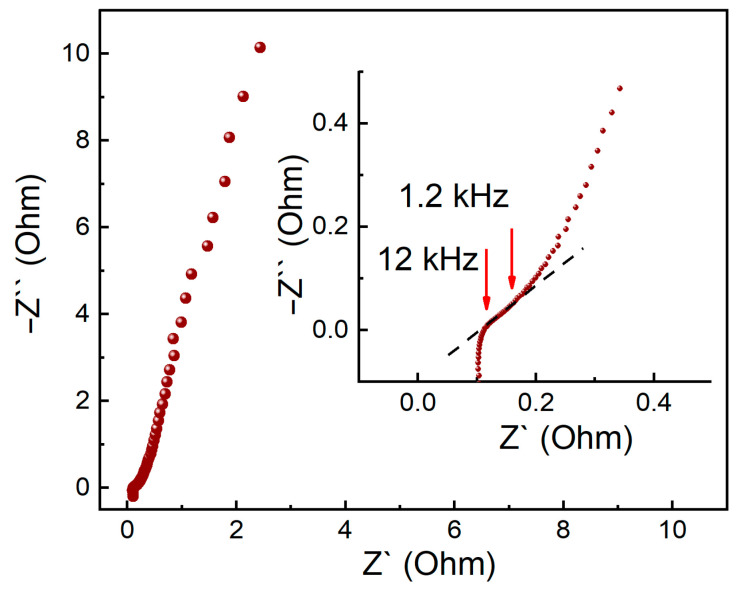
Nyquist plot of the MEA_CNT-34 sample in the humid H_2_/N_2_ systems at room temperature and atmospheric pressure; a larger area of high frequencies is shown in the inset. The points correspond to the experimentally obtained data. Red arrows indicate the beginning and end of the straight section of the graph.

Figure 3 represents the Nyquist plot used to calculate the ionic resistance of the electrode and the sum of the ionic resistance of the membrane and contacts in the electromechanical cell.

The cut-off along the axis of the real resistances (Figure 3) corresponds to the sum of the series-connected resistances of the contacts and the membrane. The dashed line in the inset shows the linear section, a projection of which on the axis of the real resistances corresponds to 1/3 of the ionic resistance of the electrode [26,27,28]. The arrows mark the section boundaries, the numbers correspond to frequencies at the start and end points.

**Figure 4:**
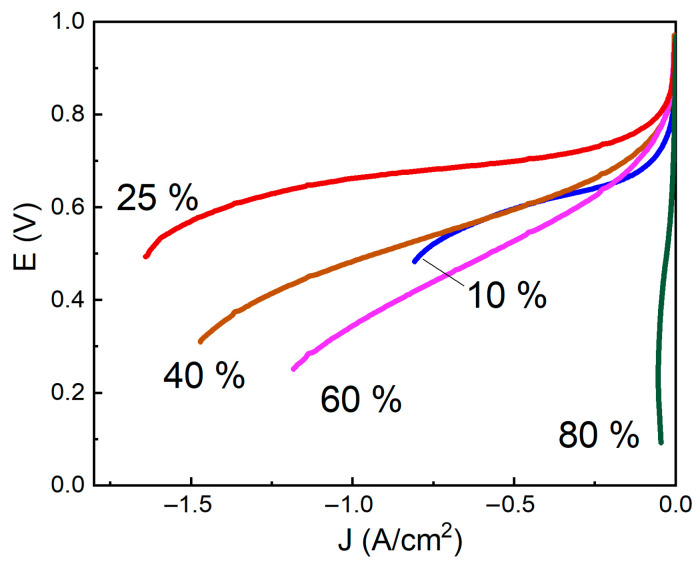
*i*–*V* curves (*iR*-free) of MEAs with various content of Nafion in the electrodes in the humid O_2_/H_2_ system, at *T* = 22 °C. Nafion content, %.

Figure 4 shows the most typical *i–V* curves of MEA samples with various content of Nafion.

The figure shows that *i*–*V* curves for MEA with various contents of Nafion differ much from each other. Initially, an increase in current density happens as the Nafion fraction increases in the electrodes, followed by a slight decrease in the current densities (at 40% and 60% Nafion), and a significant drop (80% Nafion) is observed.

Table 3 represents electrochemical characteristics of the studied samples: MEAs resistance, current density at 0.9V MA and SA of Pt in the ORR in the H_2_/O_2_ and H_2_/air systems.

**Figure 5:**
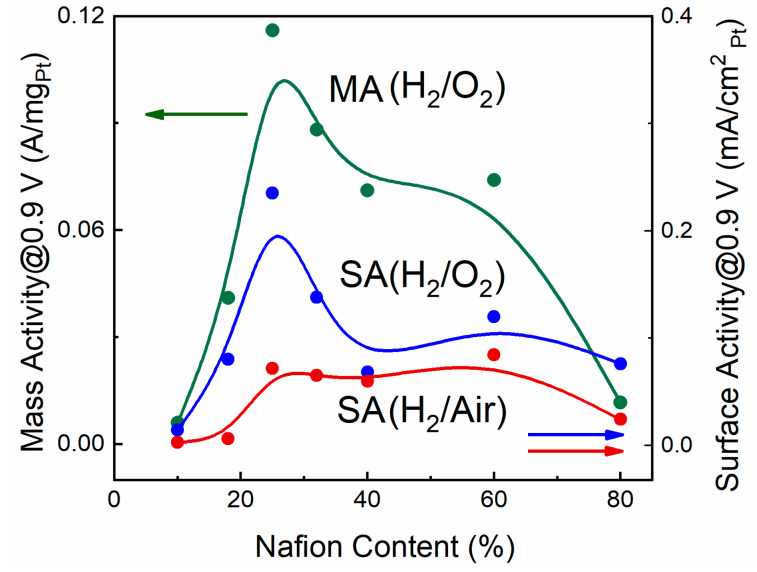
Dependencies of various types of platinum activities on Nafion content at *T* = 22 °C, atmospheric pressure, humid gases.

It can be seen from Table 3 that not only is the mass activity variable, but the surface activity also varies greatly from sample to sample. The change in MA is due to the fact that it depends on the degree of use of the platinum surface. MA is different when the ionomer/platinum ratio is too low or too high. It could be expected that the surface activity of platinum will remain more constant. It is connected to the fact that the electrochemically active platinum surface, which is measured at the electrode, participates in the ORR. If a part of the platinum surface is blocked by an ionomer (samples with a higher Nafion content), then it would seem it should not take part in the ORR, and vice versa. In this case, SA should not depend on the composition. However, experimental data suggest otherwise. Both types of activities (MA and SA) are significantly reduced in areas with low and high Nafion content. Figure 5 shows that MA drops more drastically in the area of higher ionomer contents. Both plots have a similar profile with a maximum in the area of 25% Nafion. The maximum may be explained by some structural factor that provides the optimal ionomer/platinum ratio. The very fact of SA dependence on Nafion content in the ORR indicates a defect in the measurement of electrochemically active surface of platinum or different kinetics in the case of measurement of ESA in the ORR. In addition, adsorption methods of ESA measuring will give one error or another.

Figure 5 shows dependences of platinum SA on Nafion content for two types of oxidants: oxygen and air. The plots have similar profiles. In the case of oxygen, the SA is higher (Figure 5, Table 3) due to different oxygen concentrations in these two systems and to chemical kinetics laws. A stronger difference of SA for the Nafion content of approximately 25% may be connected with the structural factors and requires a separate study. The optimal content of Nafion is determined by two competing factors. As the Nafion content decreases, the area of the three-phase region, which is necessary for the electrochemical process to occur, decreases; in addition, ionic conductivity decreases and resistive losses increase. As the Nafion content increases, the diffusion resistance increases due to the formation of a thick ionomer film.

**Figure 6:**
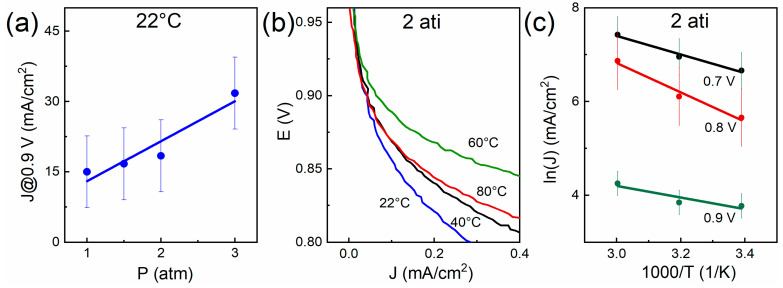
Sample MEA_CNT-34 in humid O_2_/H_2_ system: (**a**) dependence of ORR current density on pressure; (**b**) *i*–*V* curves (*iR*-free) initial sections for various temperatures; (**c**) ORR Arrhenius plot for various voltages.

Figure 6a represents the dependence of the ORR current density on pressure in the humid O_2_/H_2_ system at *T* = 22 °C for the sample with 34% CNTs. The plot is built on current densities taken at *E* = 0.9 V.

The *J*–*P* dependence (Figure 6a) can be extrapolated by the straight line. Linear approximation was derived by means of Origin Lab using the least squares method, R^2^ = 0.903. We explain relatively high extrapolation error by different MEA state for each measurement. As is shown in the figure (Figure 6a), the pressure significantly affects the platinum activity. Current density within the pressure range of 1–3 atm increases by ~3 times.

Figure 6b shows the initial sections of *i*–*V* curve for various temperatures. As the temperature increases, the current density at constant *E* increases as the temperature rises to *T* = 60 °C and decreases as the temperature increases further. The *i–V* curve at *T* = 80 °C is lower than at *T* = 60 °C. We explain this contradictory fact by the lack of sample humidity at high temperatures. The studied sample has a high (73%) porosity; as a result, the mass transfer processes (water evaporation) are intense in it. The proton conductivity of Nafion (both the membrane and the ionomer in the electrode) declines with decreasing humidity [29,30].

Figure 6c represents the Arrhenius plots for the ORR for various potentials. The slope ln(*J*)—1000/*T* is different for different potentials. The slope increases (tgα changes from 1.2 to 3.1) with an increase in overvoltage at the initial stage, then it drops (tgα changes from 3.1 to 2.0) with a further increase in overvoltage. These results indicate that as the potential decreases, the proportion of the ORR kinetic current changes, as does the concentration overvoltage contributes (at low (0.8, 0.7 V) potentials). At relatively high potentials (0.9 V), the influence of parasitic currents is possible because of the OCV proximity.

In our case, the maximum registered MA and SA of platinum with 34% CNTs at *T* = 60 °C and excessive pressure of *p* = 2 atm was 0.46 A/mg and 0.72 mA/cm^2^ at 0.9 V, respectively. At a temperature of 80 °C, the indicators drop, which, in our opinion, is due to high porosity of the electrodes and to loss of the significant part of water (see above). The obtained results of MA and SA values are higher than those of the commercial E-TEK catalyst and on par with the results obtained in recent studies.

## 4. Conclusions

In this work, MA and SA of platinum are studied in a structurally new electrode material containing platinized carbon black and CNTs. The results show that the presence of CNTs electrically coupled to Pt increases the activity of platinum in the ORR. The resulting platinum activity values exceed the DOE 2025 targets. MA and SA of platinum in the ORR in MEA with the electrodes with CNTs depend on the ionomer/platinum ratio. At the same time, both dependences have a maximum at the level of the 25% Nafion fraction. The maximum appears as a result of an optimal structure formation, which ensures the fullest use of the platinum surface and minimal concentration overvoltages. Both MA and SA of platinum depend on the Nafion fraction in the electrode. A sharp decrease in activities happens in areas with a very small (10%) and large (80%) fraction of Nafion. It relates to the structural factor; at low contents, Nafion does not ensure the full use of the platinum surface; at high contents, Nafion blocks part of the platinum surface.

The platinum activities when using oxygen and air at the anode side have similar dependencies on the Nafion fraction; with that, the activities are lower in the case of air use due to lower content of oxygen.

Arrhenius plots for potentials within the range of 0.9–0.7 V have different slopes. This is due to the influence of concentration overvoltages in the area of relatively low potentials (0.7 V) and currents of parasitic reaction at high ones (0.9 V).

MA and SA of platinum for the sample with 34% CNTs at *T* = 60 °C and excessive pressure of *p* = 2 atm amount to 0.46 A/mg and 0.72 mA/cm^2^, respectively. At a temperature of *T* = 80 °C, indicators drop, which is due to the high porosity of the electrodes and the loss of a significant part of the water.

## Figures and Tables

**Table 1:** Content of Taunit MD CNT impurities before and after treatment in nitric acid (EDX data).

Element	Before Treatment, %	After Treatment, %
C	97.4	96.1
O	1.66	3.86
Al	0.46	Not detected
Mg	0.45	Not detected
Sum	99.97	99.96

**Table 2:** Features of samples.

Sample	Share, %			*G_Pt_*, mg/cm^2^	Porosity, %
	CNTs	Pt/C	Nafion		
MEA_CNT-45	45	45	10	0.1	78.4
MEA_CNT-41	41	41	18	0.13	77.2
MEA_CNT-37.5	37.5	37.5	25	0.10	77.4
MEA_CNT-34	34	34	32	0.17	73.4
MEA_CNT-30	30	30	40	0.10	68.0
MEA_CNT-20	20	20	60	0.12	52.1
MEA_CNT-10	10	10	80	0.12	11.9

**Table 3:** Electrochemical activity (MA and SA) of platinum in the ORR in the H_2_/O_2_ system and in the H_2_/air system, at *T* = 22 °C.

Sample	R_MEA_ (Imp), Ohm	J at 0.9V (*iR*-Free), mA/cm^2^		MA, at 900 mV (*iR*-Free) A/mg (Pt)		SA, at 900 mV (*iR*-Free) mA/cm^2^ (Pt)	
		H_2_/O_2_	H_2_/Air	H_2_/O_2_	H_2_/Air	H_2_/O_2_	H_2_/Air
MEA_CNT-45	0.585	0.595	0.119	0.00595	0.00119	0.0145	0.00290
MEA_CNT-41	0.305	5.33	0.416	0.041	0.0032	0.0803	0.00626
MEA_CNT-37.5	0.295	11.6	3.55	0.116	0.0355	0.235	0.0719
MEA_CNT-34	0.219	15.0 78.6 at 60 °C	7.11	0.0882 0.462 at 60 °C	0.0418	0.138 0.721 at 60 °C	0.0652
MEA_CNT-30	0.204	7.11	6.22	0.0711	0.0622	0.0684	0.0598

## Data Availability

The data presented in this study are available on request from the corresponding author.
